# Retinoic Acid Is Involved in the Metamorphosis of the Anal Fin into an Intromittent Organ, the Gonopodium, in the Green Swordtail (*Xiphophorus hellerii*)

**DOI:** 10.1371/journal.pone.0077580

**Published:** 2013-10-25

**Authors:** Nils Offen, Ji Hyoun Kang, Axel Meyer, Gerrit Begemann

**Affiliations:** 1 Lehrstuhl für Zoologie und Evolutionsbiologie, Department of Biology, University of Konstanz, Konstanz Germany; 2 Lund Strategic Research Center for Stem Cell Biology and Cell Therapy, Department of Laboratory Medicine, Lund University, Lund, Sweden; 3 Konstanz Research School Chemical Biology, University of Konstanz, Konstanz, Germany; 4 Developmental Biology, University of Bayreuth, Bayreuth, Germany; Laboratoire Arago, France

## Abstract

In poeciliid fish the male anal fin has been transformed into a gonopodium, an intromittent organ required for internal fertilization. Elevated testosterone levels induce metamorphosis of a subset of anal fin rays to grow and form the specialized terminal structures of the gonopodium. The molecular mechanisms underlying these processes are largely unknown. Here, we investigated whether retinoic acid (RA) signaling is involved in gonopodium development in the swordtail *Xiphophorus hellerii*. We showed that *aldh1a2*, a RA synthesizing enzyme, and the RA receptors, *rar-ga* and *rar-gb*, are expressed in anal fins during metamorphosis. *aldh1a2* expression is regulated by testosterone in a concentration-dependent manner and is up-regulated in both hormone-induced and naturally developing gonopodia. *Androgen receptor* (*ar*), a putative regulator of gonopodial development, is co-expressed with *aldh1a2* and the RA receptors in gonopodial rays. Importantly, experimental increase of RA signaling promoted growth of the gonopodium and increased the number of new segments. Based on gene expression analyses and pharmacological manipulation of gonopodium development, we show that the RA signaling pathway is activated in response to androgen signaling and promotes fin ray growth and development during the metamorphosis of the anal fin into the gonopodium.

## Introduction

**Figure 1:**
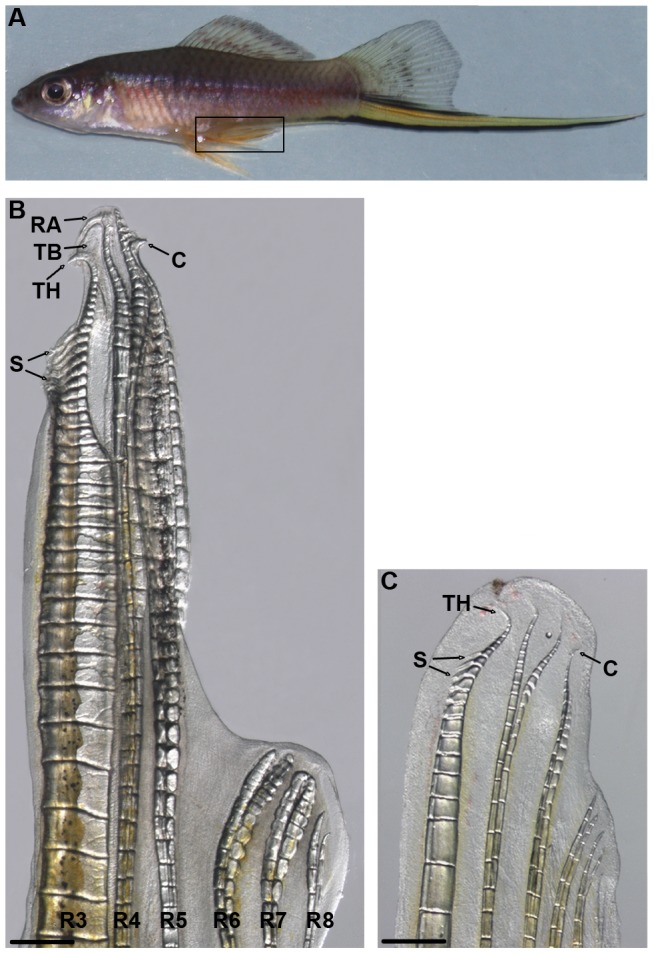
Structure of the male gonopodium. In adult swordtail males the anal fin forms a gonopodium (bracket, A). A naturally developed gonopodium with typical terminal structures that form distal lepidotrich segments (B) and a testosterone-induced gonopodium (24 days of testosterone treatment) (C); caudal is to the top and ventral to the left (B, C). Arrows indicate terminal structures. Abbreviations: C, claw; RA, ramus; TB, terminal blade; S, spine; TH, terminal hook. Scale bars: 500 µm.

A majority of all extant fish species (> 95%) belongs to the group of ray finned fish. Given more than 23,000 species the diversity of this group represents approximately 50% of all living vertebrates. Most of these fishes (98%) exhibit an oviparous mode of reproduction, while in at least 54 fish families ‘viviparous’ reproduction exists [1]. One of those families are the Poeciliid fish (Family: Poeciliidae), which consist of the three subfamilies, Aplocheilichthyinae, Procatopodinae and Poeciliinae [2]. The Poeciliinae is one of the three groups within the toothed carps (suborder Cyprinodontoidei) that are thought to have independently evolved internal fertilization and a specialized intromittent organ [3]. The intromittent organ found in poeciliid fish, called ‘gonopodium’ is sexually dimorphic and develops from the anal fin rays 3–5, the so-called 3–4–5 complex, during sexual maturation [4], [5]. These rays are a modified structure in terms of ray length, segment thickness and distal structures like blades, claws, spines, hooks and serrae [6]. The morphological characteristics of the gonopodium, particularly its terminal structures, greatly vary across species and thus have extensively been used for phylogenetic analysis [7]. By comparing the three gonopodial rays and their distal structures among several species [6], Gordon and Rosen (1951) studied the species-specific variability in the gonopodium morphology of *Xiphophorus* species including the green swordtail (*X. hellerii*) (see Fig. 1A). While ray 3 is not bifurcated, rays 4 and 5 bifurcate into two sister rays, an anterior (a) and a posterior (p) parts (Fig. 1B). Spines or serrae are formed by the rays 3 and 4p and the number of those is quite variable at the inter- and even intra-specific levels. In addition, ray 3 exhibits a terminal hook. Ray 5a carries a terminal claw that is usually present, but completely lost in some species. Furthermore, a terminal blade develops between the rays 3 and 4a in a species-specific manner in the genus *Xiphophorus*. The growth and segmentation rates can differ between the gonopodial rays, which results in segments of different lengths during the gonopodium development [7]. In general, the final length of the gonopodium depends on the body size of the individual fish [7].

Metamorphosis in male swordtails (genus *Xiphophorus*) transforms not only the anal fin into a gonopodium, but also the caudal fin into a “sword”, which is modified from ventral caudal ray 7 to 10 [8]. It has been suggested that the genetic network underlying the development of the sword is partly co-opted from the evolutionarily older gonopodium [9]. The sword evolved and was lost again secondarily in the lineage of the genus *Xiphophorus* [10], [11] whereas the 3–4–5 complex of the gonopodium evolved once in the common ancestor of the poeciliid fish. The genetic network that regulates gonopodium development remains poorly understood. Androgen signaling has been suggested to play a role in gonopodium development in *Gambusia affinis* and *X. maculatus*, since exogenous testosterone can induce gonopodium formation in juvenile fish [12], [13]. In 1941, Turner postulated a two-phase model for gonopodium development [13], [14]: first, when the testis start to mature, low levels of testosterone are released and promote outgrowth of the gonopodial rays to form the 3–4–5 complex. As the amount of hormone released increases, the differentiation of the terminal structure occurs at the growing gonopodium in a site-specific manner and in a typical temporal sequence [15]. Gonopodial development can be induced in juvenile fish by the application of exogenous testosterone; however, there are differences between naturally developing and artificially induced gonopodia [16]–[18]. Induced gonopodia basically form all terminal structures, yet they are shorter and less differentiated (Fig. 1C). It would be likely that high levels of testosterone induce fin ray growth followed by the terminal modification of fin rays at the same time [12], [13].

Independent support for the role of androgen signaling in gonopodium development is based on a study by Ogino and colleagues [19]. They found that both *androgen receptor a* and *b* are expressed in the developing gonopodium of *Gambusia affinis* and inhibition of AR signaling with flutamide perturbs gonopodium development. In addition, they identified *shh* as a target gene that is regulated by androgen signaling. *shh* and its receptor *ptc1* are expressed during gonopodium development of *G. affinis* and inhibition of *shh* signaling also blocks gonopodium development [19]. Moreover, *fgfr1* and *msxC* are up-regulated in growing gonopodial rays and are thought to promote gonopodium outgrowth [9], [20]. Experiments by Pickford and Atz (1957) suggest a putative role of thyroid signaling. Treatments of juvenile fish with thyroid hormone resulted in anal fin ray growth [21]. Environmental estrogenic biochemical can also induce gene expression of the estrogen receptor in the anal fin in medaka [21], [22].

Retinoic acid (RA), a small lipophilic and diffusible chemical, is an important signaling molecule for embryonic development because it is known to be involved in many key developmental processes, such as somitogenesis, left-right asymmetry formation, heart development and neurogenesis [23]–[27]. It is synthesised by a group of retinaldehyde dehydrogenases (Aldh1as) and stimulates gene expression through their binding to two types of receptors, retinoic acid receptors (RARs) and retinoic X receptors (RXRs) [28]. RA also plays a crucial role in the formation of paired appendages since it is essential for forelimb bud initiation in either mouse or zebrafish [29]–[34]. Furthermore, it is involved in proximo-distal patterning of skeletal elements in later stages of limb development [31], [35], [36]. Furthermore, the morphogenic effect of RA signaling (i.e. patterning formation) was found in several fin regeneration studies [37]–[40]. Recently it was also shown that RA signaling regulates the blastema formation, proliferation and the survival of mature blastema during zebrafish fin regeneration [41].

Given RA signaling is required for the development of paired fins and fin regeneration, we explore the potential role of RA in the metamorphosis of an unpaired anal fin into the gonopodium in a green swordtail fish, *Xiphophorus hellerii*. We cloned *aldh1a2* and two *rarg* receptors and examined their expression patterns during the gonopodium development. The levels of gene expression of *aldh1a2* were quantified in both testosterone-induced and naturally growing gonopodia. Through manipulative experiments of over-activating RA signaling, we further investigate a role of RA in outgrowth of ray and addition of new lepidotrich segments of developing gonopodia. We show that during anal fin metamorphosis of gonopodium outgrowth *aldh1a2* is co-expressed with *androgen receptors* and *aldh1a2* gene expression increases in a testosterone concentration-dependent manner, suggesting that RA synthesis might be controlled by androgen signaling.

## Materials and Methods

### Ethics Statement

All experiments involving animals were performed in accordance with the German Animal Welfare Act and were approved by the Government of Baden-Württemberg, Regierungspräsidium Karlsruhe, Germany (G-09/105, G-10/122).

### Fish stocks and maintenance

*Xiphophorus hellerii* were taken from stocks kept at the University of Konstanz. Fish were maintained on a 12∶12h light:dark cycle at 24°C in 110-litre densely planted aquaria and were fed TetraMin flakes and Artemia.

### Cloning *aldh1a2, rar-ga, rar-gb* and *androgen receptors*

cDNA fragments of *raldh2*, *rar-ga* and *rar-gb* were isolated from recombinant phage DNA, derived from the *X. hellerii* λ-phage cDNA library [42], by PCR using degenerate Primers. A 767 bp *aldh1a2* fragment was amplified by PCR using the Primers raldh2-fw1: 5′-GGI TAY GCI GAY AAR ATH CAY GG-3′and raldh2-rev1: 5′-ACR TTI GAR AAI ACI GTI GGY TC-3′. A 602 bp *rar-ga* and a 603 bp *rar-gb* fragment were amplified by PCR using the Primers RAR-fw2: 5′-TGY GAR GGI TGY AAR GGI TT-3′ and RAR-rev2: 5′-GGI CCR AAI CCI GCR TTR TG-3′.

To obtain appropriate size *rar-ga*/*rar-gb* fragments for RNA probe generation, the 3′ ends of the cDNAs were amplified from the cDNA library using PCR with the primer pairs RAR1-fw1: 5′-GGA GAG CTT GAA GAA CTG GTC-3′/lib-univ: 5′-CAC TAT AGG GCG AAT TGG CTA CCG-3′ for *rar-ga* and RAR2-fw1: 5′-GAA CTG GAG GAG CTT GTG AAC-3′/lib-univ for *rar-gb*. For *rar-ga*, a∼1,3 kb and for *rar-gb* a∼1,5 kb fragment was amplified. The PCR products were gel-purified using the QIAquick Gel Extraction Kit (Qiagen, Hilden, Germany) and cloned into the pCRII-TOPO vector (Invitrogen, Karlsruhe, Germany) for sequencing.

To obtain a fragment of both *androgen receptors* to generate an RNA probe giving a reliable signal, the phage λ-phage cDNA library was screened with DIG labelled RNA probe derived from *X. hellerii arb* and *arb* cDNA fragments [42]. 10^6^ recombinant phages were grown, transferred to nitrocellulose membranes (Nitropure 45 µm, Osmonics, Minnetonka, USA) and prepared for screening according to the ZAP-cDNA® Library Construction Kit manual (Stratagene, Heidelberg, Germany). The membranes were treated with Proteinase K (2 mg/ml) in PBS for 30 min at 37 °C, washed with ddH_2_0 and prehybridised in hybridisation buffer (50% Formamide, 5x Denhardt solution, 5× SSC, 0.1% SSC, 250 µg/ml sheared herring sperm DNA) for 1 h at 50°C. RNA probe was added and allowed to hybridise to the complementary cDNA for more than 16h at 50°C. Afterwards, membranes were washed five times for 10 min in 2× SSC with 0.1% SDS, two times at RT and three times at 42°C. After blocking unreacted binding sites on the membrane with 1% blocking agent (Roche, Mannheim, Germany) in maleic acid buffer (100 mM maleic acid, 150 mM NaCl) for 1 h, immunolabelling of hybridised probe was performed using a alkaline phosphatase coupled DIG antibody (1∶2000 in maleic acid buffer; Roche, Mannheim, Germany) for 2h. After washing several times in maleic acid buffer, the antibody detection was performed as described for in situ hybridisation [43]. The pBluescript phagemid containing the cDNA insert was excised from the λ -phage genome as described in the ZAP-cDNA® Library Construction Kit manual (Stratagene, Heidelberg, Germany).

### RNA probe synthesis and whole-mount in situ hybridisation

For *in situ* hybridisation juvenile fish were either treated with 5 µg/l 17-α-methyltestosterone for a variable number of days. The testosterone or mock treatment was repeated every fourth day. At the end of the treatment fish were anesthetized and 2/3 of the anal and 1/3 of the caudal fin were amputated using a sterile razor blade. The fins were fixed in 4% paraformaldehyde in PBS overnight, transferred to methanol and stored at -20°C until use.

Antisense and sense RNA probes were generated using a digoxigenin labelling kit (Roche, Mannheim, Germany). Probes for *aldh1a2*, *rar-ga* and *rar-gb*, *ara* and *arb* were generated from the cDNA fragments listed above. *In situ* hybridisation on *Xiphophorus* fins were performed as described [43] with several modifications. Prehybridisation was done 4h at 68°C in formamide solution (50% formamide, 5× SSC, 0.1% Tween20, pH to 6 with 1 M citric acid). Post-hybridisation washing steps were initiated at 68°C with formamide solution. To block unspecific binding sites 0.5% blocking reagent (Roche, Mannheim, Germany) in PBT was used. Antibody incubation was done at 4°C overnight. After fixation of stained fins/blastemata, the tissue was washed twice 20 min in PBT, 20 min in ethanol/PBT (70∶30) and 20 min in 100% ethanol and stored at 4°C. The specificity of anti-sense probes was verified with sense probe experiments.

### *In situ* hybridisation on longitudinal sections

Anal fins from individuals treated with 17-α-methyltestosterone for 7 days were fixed in 4% Paraformaldehyde (Sigma-Aldrich, Munich, Germany). Longitudinal sections of 10 µm thickness were created using a Reichert-Jung Autocut 2040 Microtome and *in situ* hybridisation was performed as described [44].

### Intraperitoneal (IP) injection of dissolved RA

Up to six juvenile individuals of *X. hellerii*, aged between 3 and 6 months, were placed in a 30-litre tank and treated as follows: Both RA injected and control groups were treated with 5 µg/l of 17-α-methyltestosterone to induce gonopodium development (1 mg/ml stock solution in ethanol; Sigma-Aldrich, Munich, Germany). Induction of gonopodia is achieved with 10 µg/l testosterone [42], but, as determined in this study, the maximum response is already obtained at 5 µg/l. 20 µl of 1 mM RA (all-trans retinoic acid in ethanol; Sigma-Aldrich, Germany), dissolved in phosphate buffered saline (PBS; Sigma-Aldrich, Munich, Germany), was injected into the peritoneum and the same volume of PBS was injected in animals of the control fish. The concentration for RA is an empirical value derived and adjusted for weight from routine injections in zebrafish [41]. Testosterone treatment (day 0 and 4) and RA injection (day 2 and 6) were repeated. For analysis of morphological changes in early gonopodium development fish were anaesthetized by incubation in a solution of 0.08 mg/ml tricaine (3-aminobenzoicacid-ethylester-methanesulfonate; Sigma-Aldrich, Munich, Germany) and anal fins/gonopodia were photographed. Photographs were taken before (at day 0) and after (at day 7) the treatment using the AxioVision software v3.1 (Zeiss) and the digital camera Zeiss AxioCam MRc. The length was measured using the software ImageJ [45]. The dataset was checked for normal distribution using graphical methods (normality plot) and statistical tests (Shapiro-Wilk). A t-test or Mann-Whitney test were used to test significant difference between RA injected and control group.

### Quantitative real-time PCR (qPCR) in testosterone-induced and naturally developing gonopodia

Juvenile individuals of *X. hellerii* (n = 3) were treated with 17-α-methyltestosterone to a final concentration of 5 µg/l under the conditions described above. Anal fins (1/2) were amputated with a sterile razor blade at 7 days of treatment. Total RNA of anal fin tissue was isolated with Trizol reagent (Invitrogen, Karlsruhe, Germany). Genomic DNA contamination was removed by incubating total RNA with DNaseI (Fermentas, St. Leon-Rot, Germany). Reverse transcription was performed for each sample in a final volume of 20 µl with 200 ng of total RNA using the Superscript III reverse transcriptase (Invitrogen, Karlsruhe, Germany). Quantitative real-time PCR (qPCR) was performed with 0.4 ul of cDNA product with iQ™ SYBR® Green Supermix (Bio-Rad, Munich, Germany) using a C1000 thermal cycler combined with a CFX96 real-time PCR detection system (Bio-Rad, Munich, Germany). The gene specific primers used are shown in Table 1. qPCR were performed according to the following program: 95°C for 15 sec, followed by 45 cycles of 95°C for 15 sec, 61.3°C for 30 sec and 72°C for 30 sec, then 95°C for 10 sec as a final step. The fold-change in expression of *aldh1a2* was analyzed using the ΔΔCt method, with β-actin as internal control [46].

**Table 1:** Gene specific primers used for qPCR.

Primer Name	Sequence (5′-3′)	F or R	Locus	Amplicon size
Bact_337F	CAGTGGTTGGCGCATACTTA	F	b-actin 3′UTR-contig	208
Bact_544R	CCCCATGTTACCGTCACTTT	R	b-actin 3′UTR-contig	
AldhI_100F	GCCTCTCCACCCACATTAAC	F	*Aldh1a2*	234
AldhI_333R	GACCGAGTCTCTGAGCATCC	R	*Aldh1a2*	

Quantitative real-time PCR (qPCR) was also performed for naturally developing gonopodia. Males with a naturally developing gonopodium and females were obtained from a community tank. We classified into two different categories of males based on the developmental phase of gonopodium. “Developing” gonopodia (n = 4) was defined as thickened and extended ray and the number of segments at ray 3 were between 10 and 34. “Almost-mature” gonopodia (n = 8) was defined when differentiated distal structures were present, ray 3 had developed more than 35 segments, and a colored sword was visible in the caudal fin. Females (n = 5) were used as a control group; the number of ray 3 segments was 8 or 9 in all individuals.

The tip of the gonopodium (4 mm) was cut from each individual used for total RNA isolation. RNA preparation, synthesis of cDNA and qPCR were performed as described above. One-way ANOVA was followed by post hoc testing to test significant difference of the level of *aldh1a2* gene expression among different concentrations. Linear regression was performed to predict the effect of testosterone on gene expression levels. Delta Ct was used as dependent variable. A p-value lower than 0.05 was considered to be significant.

### Phylogenetic analysis and protein domains

cDNA sequences of retinoic acid receptors, *aldh1a* enzymes and androgen receptors were sampled from GenBank and Ensembl using the Blast algorithm [47] and aligned using ClustalW [48]. For aldh1as the full cDNA alignment (excluding the third position) was used for the phylogenetic analysis. For the retinoic acid and androgen receptors a cDNA fragment coding for the C4 zinc finger and the hormone binding domain was used to build the tree. Based on the alignments, phylogenetic relationships were constructed using maximum likelihood (ML) and Bayesian methods of phylogeny inference [49]. ML analyses were performed using PHYML 2.4 [50]. The best fitting models of sequence evolution for ML were obtained by ModelTest 3.7 [51]. For retinoic acid receptors the Tamura-Nei model TrN+I+G (alpha = 0.7916, p_inv_ = 0.4111; [52]), for aldh1a enzymes the general time reversible model GTR+I+G (alpha = 1.1961, p_inv_ = 0.2136; [53]) and for androgen receptors the Hasegawa-Kishino-Yano model HKY+G (alpha = 0.5019, T_Ratio_ = 1.1257; [54]) was used. ML tree topologies were evaluated by a bootstrap analysis with 500 replicates [55]. To confirm obtained tree topologies Bayesian analyses were initiated with random seed trees and were run for 1,000,000 generations. The Markov chains were sampled at intervals of 100 generations with a burn in of 1000 generations. Bayesian phylogenetic analyses were conducted with MrBayes 3.0b4 [56].

Conserved protein domains were identified by searching the Pfam database (http://pfam.sanger.ac.uk/search).

The following sequences were used for the phylogenetic analysis:

*aldh1a1: Homo sapiens* (NM_000689), *Mus musculus* (NM_013467), *Gallus gallus* (NM_204577), *Xenopus laevis* (NM_001087772)

*aldh1a2: Homo sapiens* (NM_003888), *Mus musculus* (NM_009022), *Gallus gallus* (NM_204995), *Xenopus laevis* (NM_001090776), *Danio rerio* (AF315691), *Gasterosteus aculeatus* (ENSGACT00000020927), *Oryzias latipes* (ENSORLT00000010445), *Takifugu rubripes* (NM_001033639), *Tetraodon nigroviridis* (CAAE01013867)

*aldh1a3: Homo sapiens* (NM_000693), *Mus musculus* (NM_053080), *Gallus gallus* (NM_204669), *Xenopus laevis* (NM_001095605), *Danio rerio* (DQ300198), Gasterosteus aculeatus (ENSGACT00000018580), Tetraodon nigroviridis (GSTENT00012805001), *Takifugu rubripes* (NEWSINFRUT00000155714)

*aldh1a1/2/3*: *Ciona intestinalis* a (ENSCINT00000016285), *Ciona intestinalis* b (ENSCINT00000016054), *Ciona intestinalis* c (ENSCINT00000016069), *Ciona intestinalis* d (ci0100136702)

*rarg: Homo sapiens* (NM_000964), *Mus musculus* (NM_009024), *Gallus gallus* (X73972), *Notophthalmus viridescens* (X17585)

*rarga*: *Danio rerio* (NM_131406), *Tetraodon nigroviridis* (GSTENT00024106001), *Takifugu rubripes* (GENSCAN00000028342)

*rargb*: *Danio rerio* (NM_131399), *Gasterosteus aculeatus* (ENSGACT00000007038), *Takifugu rubripes* (GENSCAN00000013561), *Tetraodon nigroviridis* (GWSHT00007447001)

*rarg: Homo sapiens* (NM_000965), *Mus musculus* (NM_011243), *Gallus gallus* (NM_205326), *Notophthalmus viridescens* (AY847515)

*rarg: Homo sapiens* (NM_000966), *Mus musculus* (NM_011244), *Mesocricetus auratus* (AY046945)

*rarga*: *Danio rerio* (S74156), *Takifugu rubripes* (GENSCAN00000021740), *Tetraodon nigroviridis* (GSTENT00028047001), *Gasterosteus aculeatus* (ENSGACT00000012380)

*rargb*: *Danio rerio* (NM_001083310), *Gasterosteus aculeatus* (ENSGACT00000000789), *Takifugu rubripes* (GENSCAN00000014750)

*ar*: *Homo sapiens* (NM_000044), *Mus musculus* (NM_013476), *Gallus gallus* (NM_001040090), *Xenopus laevis* (NM_001090884)

*arb: Gasterosteus aculeatus* (AY247207),(AY247206),*Oryzias latipes* (NM_001122911), Tetraodon nigroviridis (CAAE01014703), Takifugu rubripes (GENSCAN00000027349), *Oreochromis niloticus* (AB045212), *Gambusia affinis* (AB182329)

*arb: Gasterosteus aculeatus* (GENSCAN00000022206),*Oryzias latipes* (NM_001104681), *Tetraodon nigroviridis* (CAAE01014998), *Takifugu rubripes* (GENSCAN00000026438), Oreochromis niloticus (AB045211), Gambusia affinis (AB174849)

*pgr*: *Homo sapiens* (NM_000926), *Mus musculus* (NM_008829), *Gallus gallus* (NM_205262)

## Results

### Isolation of RA- and androgen signaling pathway components from *X. hellerii*

**Figure 2:**
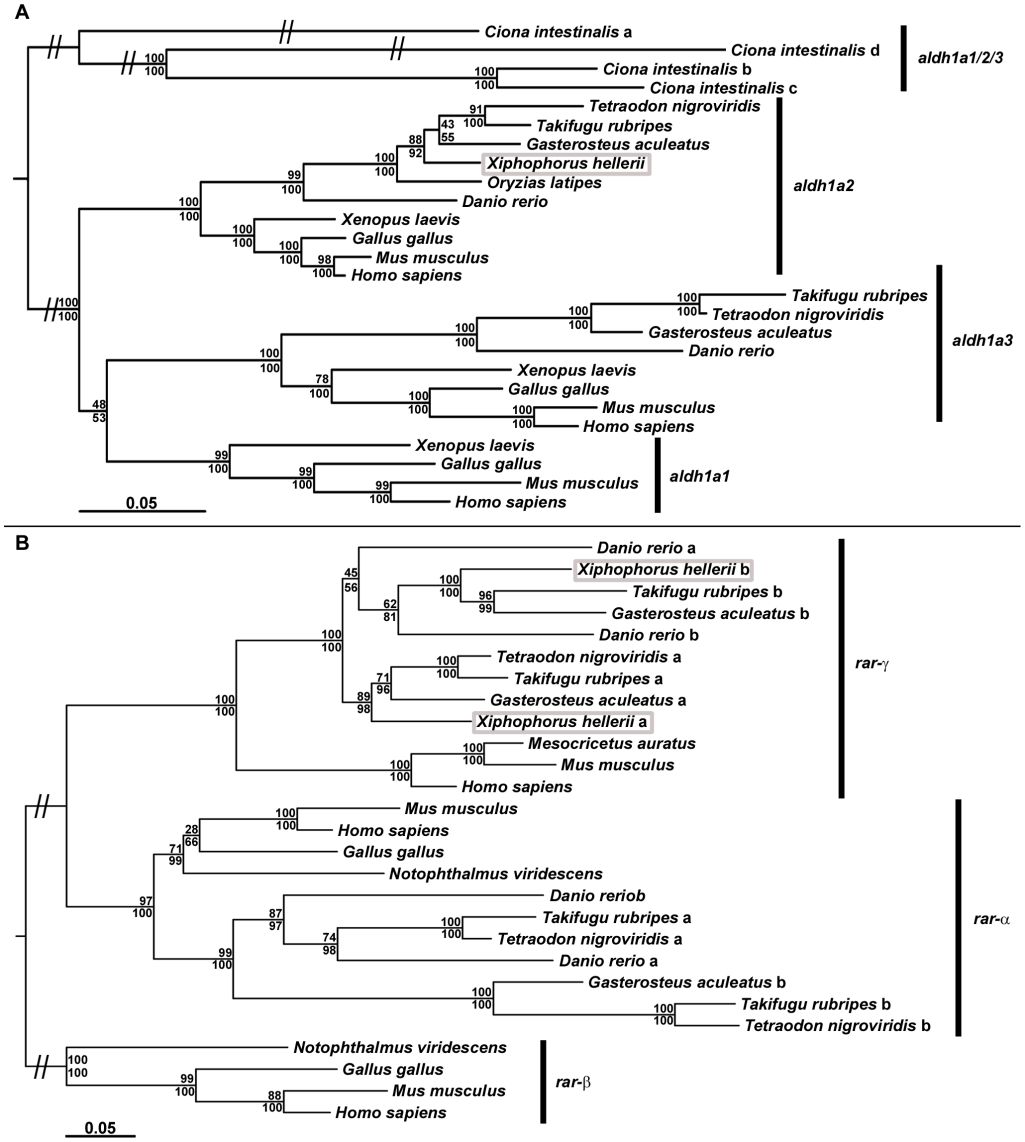
Phylogenetic reconstruction of *aldh1a* and *rar* sequences. Phylogenetic analysis of chordate *aldh1a* enzymes (A) and *retinoic acid receptors* (B) using PhyML (upper values) and MrBayes (lower values). For the analysis the coding regions of *aldh1a* and *rar* cDNAs were used. The position of the *X. hellerii* orthologs of *aldh1a2*, *rar-ga* and *rar-gb* within the two phylogenies is highlighted (grey box).

To test whether RA is involved in the development of the gonopodium, we screened a *X. hellerii* cDNA library that we had constructed from developing swords and gonopodial tissue, for orthologs of RA synthesizing enzymes (*aldh1as*) and *retinoic acid receptors* (*rars*). We amplified a 721 bp fragment of a putative *aldh1a2* ortholog (FJ372848) that codes for a 240 aa sequence of the protein. Phylogenetic reconstruction of *aldh1a* enzymes, using coding sequences, confirmed that the fragment is a partial sequence of the *X. hellerii aldh1a2* ortholog (Fig. 2A). In addition, four cDNA fragments were cloned, that encompassed parts of the open reading frames and the complete 3′-UTR sequences of two *rar-g* orthologs. Phylogenetic reconstruction of *retinoic acid receptors*, using coding sequence, confirmed that we cloned partial sequences of *X. hellerii rar-ga* (FJ372849) and *rar-gb* (FJ372850) (Fig. 2B). The partial *rar-ga* and *rar-gb* sequences code for parts of the protein including most of the zinc finger DNA binding domain and the complete nuclear hormone receptor ligand-binding domain.

**Figure 3:**
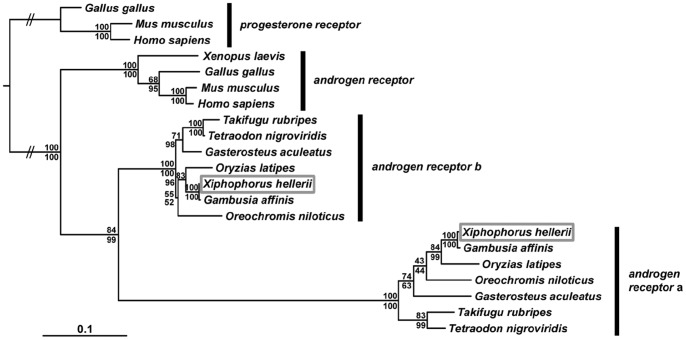
Phylogenetic reconstruction of *androgen receptor* sequences. Phylogenetic analysis of vertebrate *androgen receptors* using PhyML (upper values) and MrBayes (lower values). For analysis the coding regions of *androgen receptor* (*ar*) cDNAs were used. The position of the *X. hellerii ara* and *arb* orthologs within the phylogeny is highlighted (grey box).

To examine the expression of the androgen receptors involved in metamorphosis of the gonopodium, we screened the cDNA library by filter screening and isolated two androgen receptor cDNAs. A 2418 bp cDNA clone codes for 596 aa of *androgen receptor a*, including the zinc finger DNA binding domain and the complete nuclear hormone receptor ligand-binding domain and the 3′UTR. A second 3867 bp cDNA clone was identified as *androgen receptor b* and covers the complete coding region and 3′-UTR sequence as well as parts of the 5′-UTR sequence. We detected the same two conserved domains in the 756 aa protein sequence as in the first clone. Phylogenetic reconstruction using the amino acid sequence translations from cDNA fragments coding for the zinc finger and the ligand binding domains confirmed the cDNAs as *androgen receptor a* (FJ372851) and *androgen receptor b* (FJ372852) orthologs, respectively, of *X. hellerii* (Fig. 3).

### RA signaling pathway components are expressed in developing gonopodia

**Figure 4:**
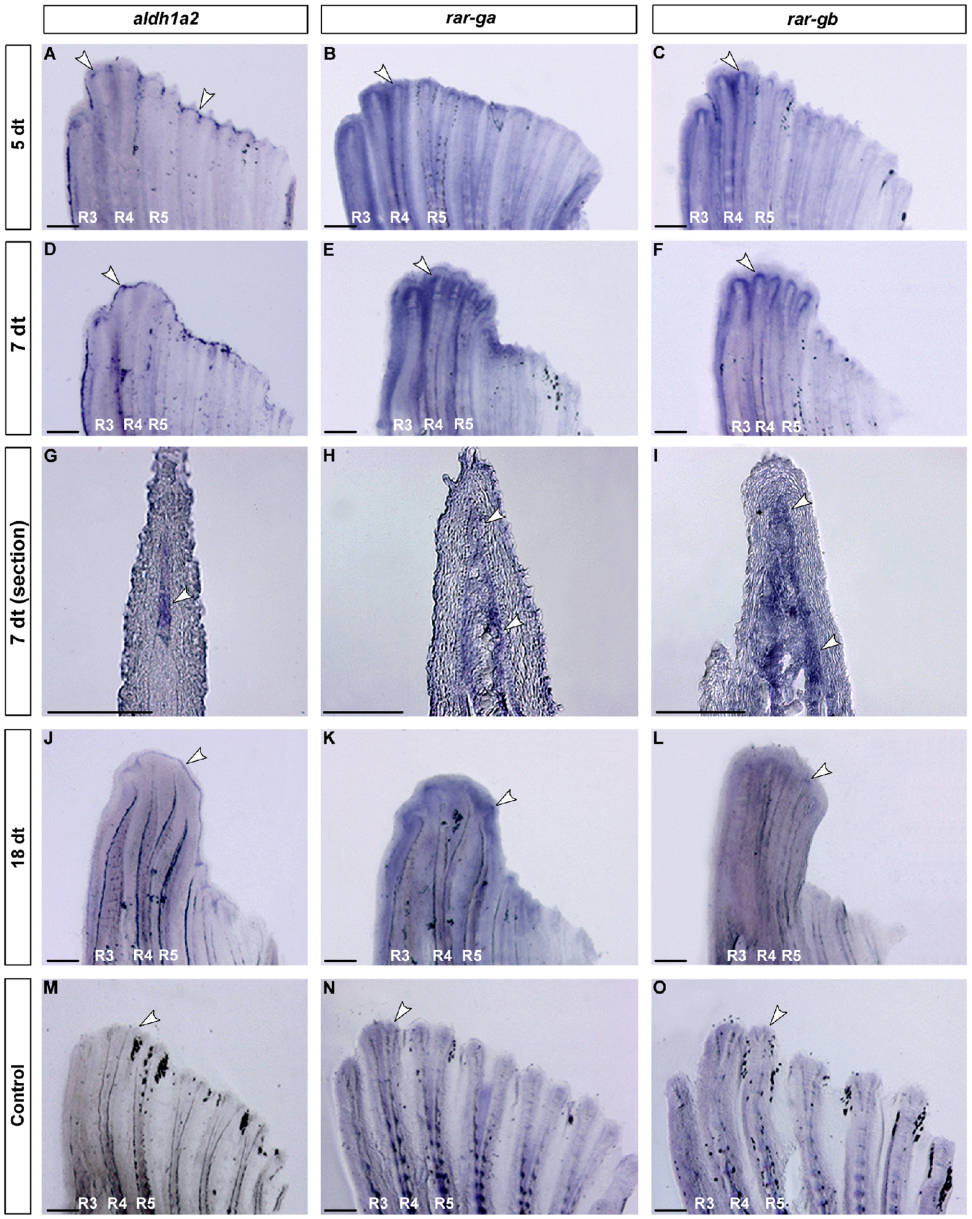
Expression of *aldh1a2*, *rar-ga* and *rar-gb* during gonopodium development. *aldh1a2* and both *rarg* paralogs are expressed in developing gonopodia of *X. hellerii*. At 5 (A), 7 (D) and 18 (J) days of testosterone treatment (dt) *aldh1a2* is expressed the distal-most mesenchyme of the main gonopodial rays and extends into rays 6 and 7 (G). *aldh1a2* expression was not detected under identical conditions in control fins (M). *rar-ga* and *rar-gb* are expressed in an overlapping pattern. At 5 (B, C), 7 (E, F), and 18 dt (K, L) strong expression of both genes could be detected in the gonopodial rays 3–5 and also partly in ray 6 and 7. At 18 dt both genes appeared slightly down-regulated. The expression domains of both genes include the distal-most and more proximo-lateral mesenchymal cells (H, I). No up-regulation was detected in the control fins (N, O). White arrowheads highlight gene expression. (n = 6 for 5 and 7dt, n = 4 for 18 dt, and n = 5 for controls for every probe); scale bars: A-F and J-O: 200 µm; G-I: 100 µm.

As a first step towards exploring whether RA signaling plays a role in gonopodium development, we analysed the expression of *aldh1a2* and the two *rar-*paralogs. To induce metamorphosis of the pre-adult anal fin, we treated juvenile individuals of *X. hellerii* with 17-α-methyltestosterone and analysed gene expression by *in situ* hybridisation at different stages of gonopodium development. After 5 days of testosterone treatment (dt), *aldh1a2* (Fig. 4A), *rar-ga* (Fig. 4B) and *rar-gb* (Fig. 4C) were up-regulated in the 3–4–5 complex that gives rise to most of the gonopodial structures. In addition, the two *rars* (Figs. 4B, C) and *aldh1a2* were also expressed in fin rays 6 and 7 that flank the 3–4–5 complex (compare Figs. 4A, B, C). At 7 dt, when the elongation of the 3–4–5 complex was clearly visible, *aldh1a2* was expressed in a continuous distal stripe that encloses the distal tip of the 3–4–5 complex and several other gonopodial rays (Fig. 4D). The expression patterns of the *rarg* transcripts at 7 dt appeared to be unchanged compared to 5 dt (Figs. 4E, F). *In situ* hybridisation on fin sections revealed that *aldh1a2* was expressed in the distal-most mesenchyme of the gonopodial rays (Fig. 4G). The two *rarg* transcripts showed overlapping expression with *aldh1a2* in the distal mesenchyme; however, we detected additional expression domains in the lateral mesenchyme (Figs. 4H, I). Both receptors did not seem to differ in their temporal-spatial expression. At later stages of gonopodium development (18dt), when the first terminal structures, the spines, had started to form, *aldh1a2* remained up-regulated (Figs. 4J, C). *rar-ga* and *rar-gb* were slightly down-regulated compared to 7 dt (Figs. 4K, L). In untreated control fins no up-regulation of *aldh1a2* and *rar-g*s was detected (Fig. 4M-O). The two *rar-gs* were expressed at a basal level (Figs. 4N, O), whereas *aldh1a2* expression was not detected by *in situ* hybridization (Fig. 4M). The observed changes in gene expression profiles during gonopodial development suggest that transcriptional activation of *aldh1a2* and the two *rar-g* paralogs correlates with the transformation of the male anal fin into an intromittent organ.

### Androgen receptors are expressed in developing gonopodia

**Figure 5:**
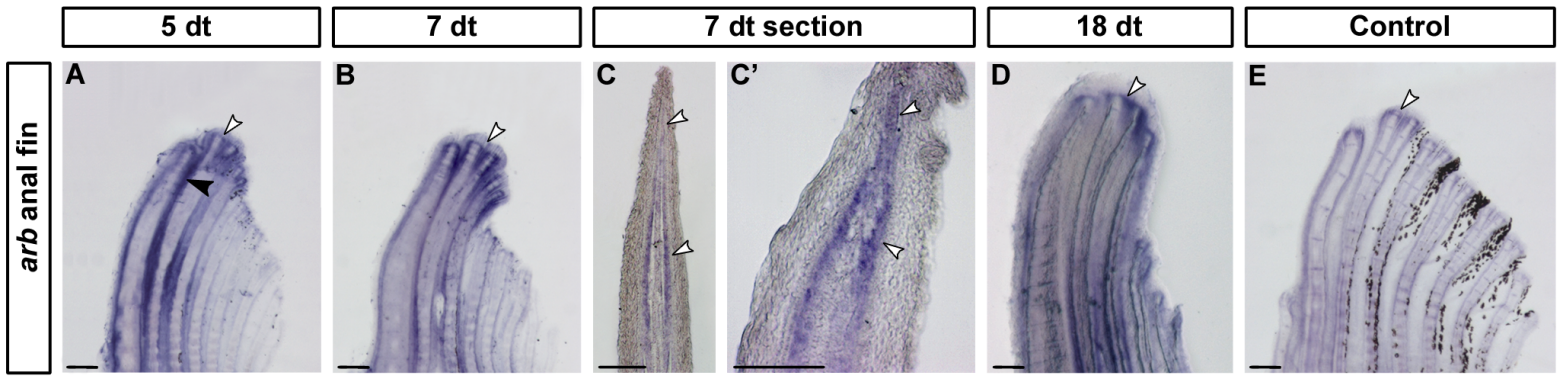
Expression of *androgen receptor b* in the developing gonopodium. *androgen receptor b*(*arb*) is expressed in developing gonopodia of *X. hellerii*. At 5 days of testosterone treatment (dt) *arb*is strongly up-regulated in distal tip of the gonopodial rays 3, 4 and 5 and clearly weaker in the rays 6–7 (A). Expression could also be detected in the inter-ray tissue (black arrowhead). This expression pattern persists at 7 (B) and 18 dt (D). Longitudinal sections of anal fins after 7 dt revealed *arb* to be expressed both in the distal and proximal mesenchyme (C, C'). In control fins *arb* is expressed at basal levels (E). White arrowheads indicate gene expression. (n>10 for every stage and probe; scale bars: A, B, D–F and H, I: 200 µm; C, C', G: 100 µm).

*Androgen receptor a* showed a diffuse pattern of low-level expression in all fin rays of the developing gonopodium (data not shown). In contrast, after 5 days of testosterone treatment (dt) *androgen receptor b* (*arb*) was strongly up-regulated in the distal tip of the 3–4–5 complex and in the inter-ray tissue (Fig. 5A). After 7 dt, when the 3–4–5 complex was clearly elongated, a similar pattern was detected that differed from the low-level expression in the fin rays that do not participate in gonopodium formation (Fig. 5B). *In situ* hybridisation on sections of 7 dt gonopodia revealed that *arb* was expressed in a layer of mesenchymal cells underlying the epidermis (Fig. 5C). The expression domain covers the distal-most mesenchyme, where also *aldh1a2* is expressed, and expands proximally, overlapping with the expression of the two *rar-g* paralogs (compare Figs. 4G–I and 5C). Up-regulation of *arb* in the 3–4–5 complex persisted in later stages of gonopodium development (18 dt) when the first distal structures were present (Fig. 5D). The expression of *arb* could also be detected in the 3–4–5 complex of untreated control fins, but at lower levels than in treated fin (compare Fig. 5A to 5E). In addition, expression could be detected in the segment borders when samples were stained for a longer time period (Fig. 5E).

### Induction of *aldh1a2* expression is proportional to the rate of metamorphosis

**Figure 6:**
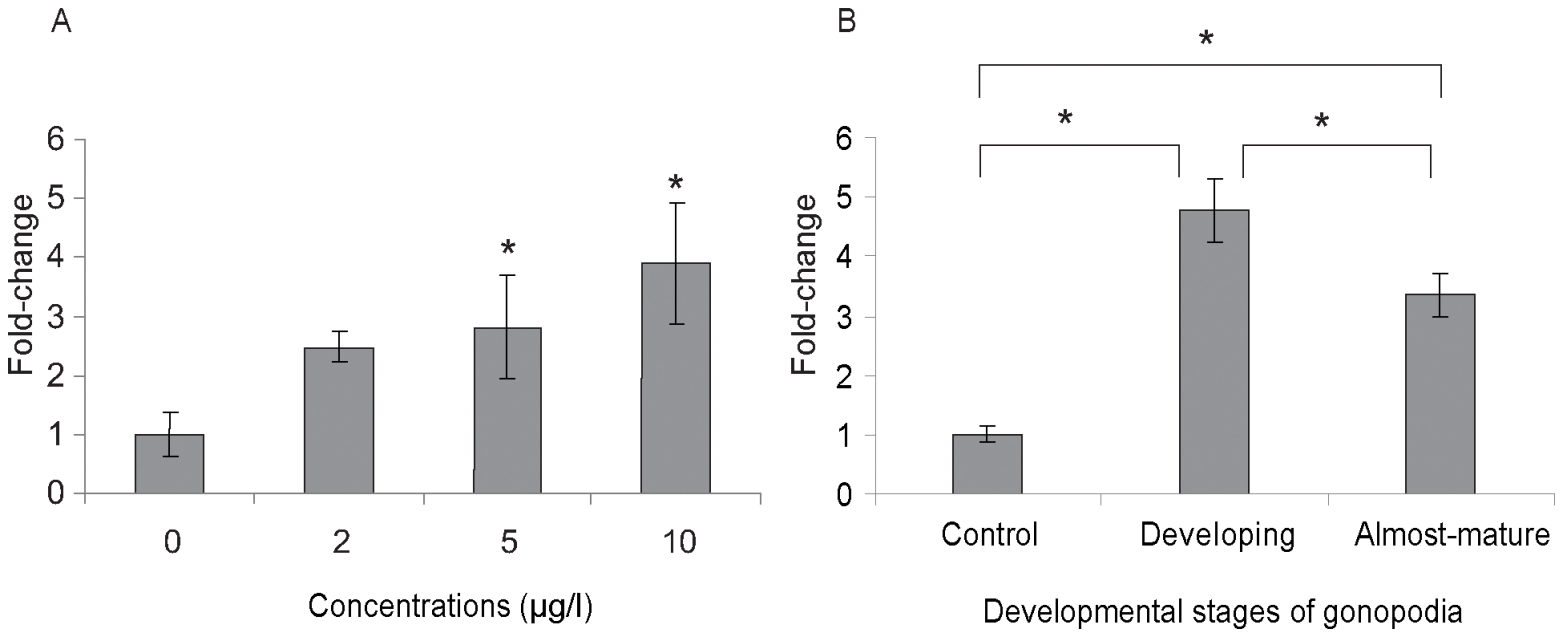
Increase in *aldh1a2* expression in testosterone-induced gonopodia. Fold-changes of *aldh1a2* expression in testosterone-induced gonopodia after 7 days of treatment (A) and in naturally developing gonopodia (B). Anal fins of females (n = 5) were used as control group and developing (n = 4) and complete (n = 8) gonopodia from males were used (B). Asterisks indicate statistically significant differences compared to the control (p<0.05, ANOVA). Figure is plotted as means ± SE.

**Figure 7:**
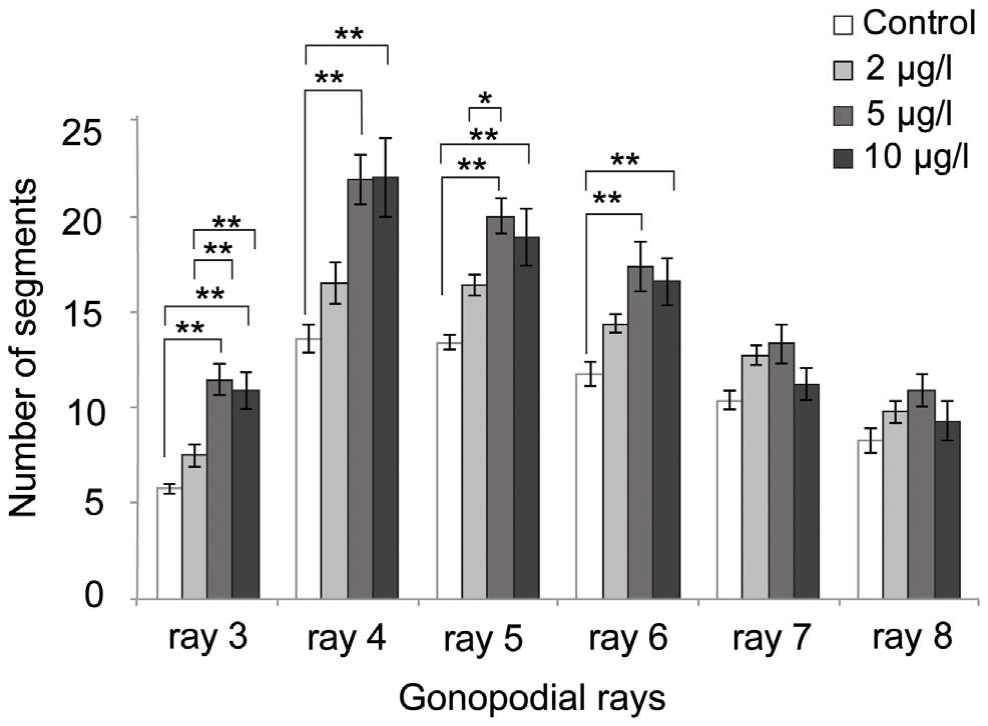
Segment numbers of rays 3, 4, 5, 6, 7 and 8 in testosterone-induced gonopodia at day 7 during treatment. The number of segments of each ray in different concentrations of 17-α-methyltestosterone was measured (n = 7 to 8). Asterisks indicate statistically significant differences (*p<0.05, **p<0.01, rank by Tukey). Figure is plotted as means ± SE.

To better understand the dynamics of RA signaling during the metamorphosis of the anal fin towards a gonopodium we examined the regulation of *aldh1a2* expression by real-time quantitative PCR (qPCR) (Fig. 6). To test whether *aldh1a2* is induced by testosterone in a concentration-dependent manner, we investigated the response to increasing concentrations (2, 5 and 10 µg/l) of 17-α-methyltestosterone. The amount of transcript of *aldh1a2* after 7 days of treatment was increased at all three concentrations of testosterone-treated fish compared to mock-treated controls (Fig. 6A). The concentrations of 5 and 10 µg/l displayed 2.8- to 3.9-fold induction in *aldh1a2* expression (p<0.05, ANOVA), whereas the concentration of 2 µg/l showed 2.5-fold induction with moderate p-value (p = 0.052, ANOVA). Linear regression analysis based on the qPCR analysis confirmed the association of the concentration of 17-α-methyltestosterone with the level of *aldh1a2* gene expression (r^2^ = 0.461, p = 0.015) and supports the notion that *aldh1a2* expression levels correlate with an increase of androgen signaling. In addition, we observed that the higher testosterone concentrations (5 and 10 µg/l) induced more developed gonopodia in terms of the average number of segments that have developed in rays of the 3–4–5 complex when compared to the low concentration (Fig. 7). Thus under our experimental regime the maximum number of segments is already induced at a concentration of 5 µg/l, whereas a further increase in concentration has no additional effects on segment number.

### Differentiation of terminal gonopodial ray structures correlates with decreased RA signaling

The development of an artificially induced gonopodium differs from that of a naturally developing gonopodium in that exogenous addition of testosterone leads to a shorter overall length of the gonopodium (Fig. 1) [18]. Therefore, *aldh1a2* gene expression was also investigated in naturally developing gonopodia (Fig. 6B). Because of large variation and unpredictability in the maturation times in different individuals it was technically impossible to obtain large numbers of males at the exact same stages of natural gonopodium development. Hence, we introduced two categories for the stage of gonopodium development, which we classified as “developing” gonopodia (apparent increase in the length of the 3–4–5 complex) and “almost-mature” (formation of distal structures) gonopodia, as compared to the anal fins of mature females as a control (see details under Experimental Procedures). *aldh1a2* transcripts were considerably increased both in “developing” gonopodia (by 4.8 times) and in “almost-mature” gonopodia (by 3.7 times) (Fig. 6B). However, *aldh1a2* expression levels are significantly higher in “developing” compared to “almost mature” gonopodia (p<0.05, ANOVA). These results suggest that *aldh1a2* gene expression is up-regulated in naturally developing gonopodia and is then down-regulated towards completion of the final gonopodial structure.

### RA signaling is involved in patterning of the gonopodium

The positive correlation between increases in testosterone levels and *aldh1a2* expression suggested that RA signaling might be a major determinant of gonopodial development. To test this idea more directly, we enhanced RA signaling during testosterone-induced metamorphosis and examined its effects on gonopodium development. *Xiphophorus hellerii* juveniles were treated twice with 17-α-methyltestosterone to induce gonopodium development, followed by intraperitoneal (IP) injection of *all-trans* RA on days 4 and 6 (see Materials and Methods). For the experiment we chose a 17-α-methyltestosterone concentration of 5 µg/l, which we found to be the lowest concentration that led to the maximum increase in segment number. The growth of gonopodial rays was compared between controls (PBS–injected) and RA-injected fish at day 7.

**Figure 8:**
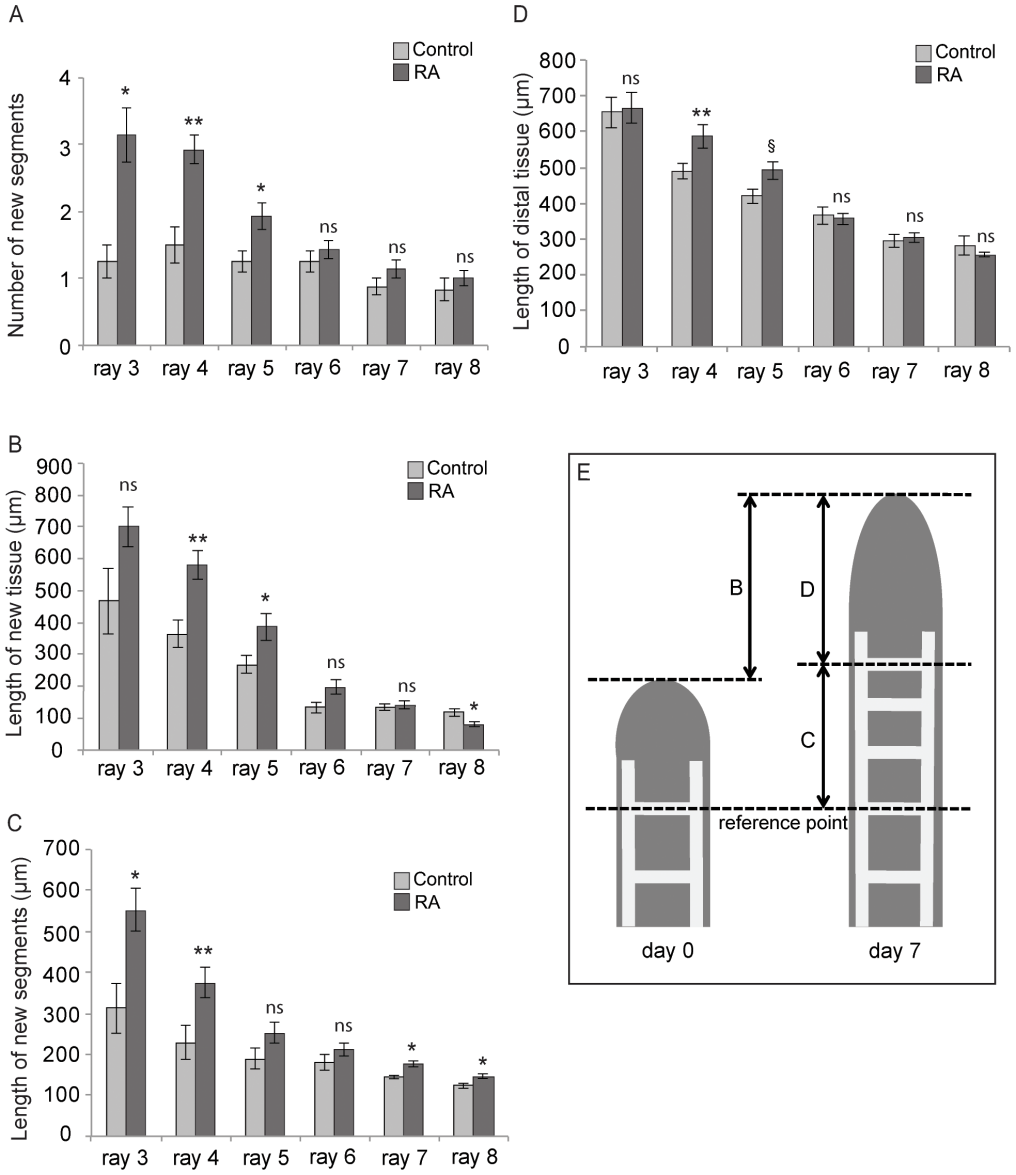
Increased activation of RA signaling in testosterone-induced gonopodia affects growth and development. The number of new segments (A) and the length (in μm) of the tissue added by growth (B) in artificially induced gonopodia were compared in control and RA-injected fish after 7 days of treatment (n = 4 for control and n = 7 for RA). For these measurements, we initially set a reference point, which is the distal most segmental border of the last formed segment at day 0 (before treatment) (E). The distance from the reference point to the tip of developing gonopodia at day 0 was excluded from the same distance at day 7 (B). The total distance at day 7 in each ray was significantly increased in RA-injected fish (B). The length of new segments (from the reference point to the distal boundary of the last segment at day 7) (C) and the length of tissue of tip of gonopodia (from the distal border of the last segment to the tip of gonopodia at day 7) (D) were measured separately. Both lengths (C, D) were contributed to the increased total length of outgrowth of gonopodia (B). *indicates statistically significant differences compared to the control and § indicates marginal significance (*p<0.05, **p<0.01, 0.05<^§^p<0.08). Figure is plotted as means ± SE.

First, we found that the total number of new segments was significantly higher in RA-injected fish relative to control fish in gonopodial rays of the 3–4–5 complex (2.49 segments in RA-injected vs. 1.35 in control, Mann-Whitney, p<0.05) (Fig. 8A). This increase in segment number is specific for the 3–4–5 complex and was not observed for non-gonopodial rays (rays 6, 7 and 8). Second, the amount of tissue added to each ray as a result of proliferation was increased in gonopodial rays in RA-injected fish. While the lengths of rays 4 and 5 were significantly increased in RA-treated fish compared to control animals (t-test, Mann-Whitney respectively, p<0.05), we found only marginally significant differences in the length of ray 3 (t-test, p = 0.073). The length of the non-gonopodial rays 6 and 7 was not significantly increased and that of ray 8 indeed was decreased in RA-injected fish (t-test, p<0.05) (Fig. 8B). To examine the relative contributions of segmented rays and unsegmented distal tissue to the new tissue added by growth, we measured the former as distance between a reference point and the distal-most segmental border (Fig. 8C) and the latter as distance between distal-most segmental border and the tip of the ray (Fig. 8D; see diagram in Fig. 8E). The total length of newly formed segments (Fig. 8C) was significantly increased for rays 3, 4, 7 and 8 in RA-injected fish compared to control fish (t-test, p<0.05, p<0.01 respectively). It was increased significantly more in gonopodial rays (rays 3 and 4) than non-gonopodial rays (rays 7 and 8) (Fig. 8C). No significant differences, however, were found in rays 5 and 6 between the groups (Fig. 8C). The length of the distal region was also increased for rays 4 and 5 (t-test, p<0.05 and p = 0.059 respectively) (Fig. 8D). This shows that both areas contributed to the increased total length of outgrowth (proliferation) of gonopodia in the RA-injected fish. Overall, an increase in RA signaling during anal fin metamorphosis increases the rates of distal tip proliferation and proximal ray segmentation and suggests that gonopodial development is driven by *aldh1a2*-dependent RA signaling.

## Discussion

Although the role of RA signaling in embryonic development and fin regeneration has been studied in several groups of organisms [25], [57], [58], its role in fin metamorphosis of teleost fish had remained largely unexplored. Gonopodium development differs from embryonic fin development and regeneration in several aspects. First, the gonopodium forms during sexual maturation when its development is triggered by increased synthesis of sex steroids. Second, the gonopodium is a male specific structure that does not develop in females under natural conditions. Third, a specific subset of gonopodial rays develops into a morphologically highly specialized structure with a prominent proximal-distal polarity. Therefore, an investigation of the role of RA signaling pathway in developing gonopodia should provide valuable information for the understanding of its role in postembryonic metamorphosis of fins and the evolution of the gonopodium in poeciliid fish. The current study provides functional evidence that RA signaling is involved in anal fin metamorphosis in the green swordtail, *X. hellerii*.

### Up-regulation of *aldh1a2* in hormone-induced gonopodia suggests that RA signaling is involved in gonopodium development

Our expression analyses showed that RA signaling pathway components (*aldh1a2* and the two *rar-g* paralogs) are continuously expressed in testosterone-induced gonopodia at different time points (5, 7 and 18 dt) (Fig. 4). Expression of *aldh1a2* was up-regulated by testosterone in a concentration-dependent manner (Fig. 6A). These results suggest that RA signaling plays a role in gonopodium development. It is worth noting that, due to high levels of exogenous testosterone, hormone-induced gonopodia show alterations in final morphology and developing timing compared to naturally developing gonopodia. Hormone induced gonopodia are shorter (compare Fig. 1B and 1C) and also possess a reduced number of segments (e.g., 20 for hormone induced ray 3 vs. 38 normal ray 3 adult male, Fig. 7) compared to naturally developing ones [12], [18]. In addition, the developmental process of hormone-induced gonopodia was completed in a much shorter time (18 days) than what is observed for naturally developing gonopodia (up to 6 months). We propose that these differences can be explained by Turner's two-phase model, in which early and late phases of gonopodium development depend on changes in internal testosterone levels. Turner predicted that low concentrations of testosterone at the beginning of maturation would induce outgrowth of the 3–4–5 complexes, whereas high concentrations induce the formation of terminal structures [59]. Under this model, terminal segments that contribute to the distal structures could develop immediately in experimentally induced gonopodia due to the premature induction of the second phase by high levels of exogenous testosterone. This view is consistent with earlier observation [12], [13] as well as our own findings (Fig. 1).

The expression of *aldh1a2* showed temporally and spatially distinct patterns: *aldh1a2* expression was stronger in rays 6 and 7 than in the 3–4–5 complex at day 5 when the gonopodium initiates outgrowth, whereas when the gonopodium is further developed, at days 7 and 18, *aldh1a2* expression is considerably stronger in the 3–4–5 complex region (Fig. 4). The rise in *aldh1a2* expression in the 3–4–5 complex therefore correlates with the accumulation of sex steroids throughout gonopodium development. In addition, a rise in testosterone causes a concomitant rise in *aldh1a2* expression. These observations suggest that the levels of RA signaling, through the regulation of RA synthesis, are controlled by rising concentrations of sex steroids during gonopodial development.

### Naturally developing gonopodia show a dynamic up-regulation of *aldh1a2* expression

Naturally developing gonopodia, in which the different phases of gonopodium development are more distinct, could help to determine the precise roles of RA signaling. The metamorphosis of the anal fin into an intromittent organ involves several developmental events such as fin ray outgrowth, segmentation of new lepidotrichs and development of specialized distal structures like serrae, spines, hooks and claws [6]. We defined two categories of naturally developing gonopodia, “developing” and “almost-mature”, that may be considered as the first (outgrowth, including addition of new segments) and second phase (formation of distal structures) in Turner's model of gonopodium development, respectively. Up-regulation of *aldh1a2* expression in both phases of naturally developing gonopodia suggests that RA signaling is involved in the outgrowth of the gonopodium in the early phase, as well as the terminalisation in the later phase (Fig 6B). Gene expression of *aldh1a2* was significantly increased when gonopodia experienced fin ray outgrowth (Fig. 6B). Elevated levels of gene expression, albeit reduced, still persisted during terminal development (Fig. 6B). Thus, dynamic expression of *aldh1a2* is associated both with fin ray outgrowth and formation of terminal structures.

### Increased RA signaling promotes gonopodium proliferation and segmental differentiation

To substantiate the idea that the correlation between the dynamics of *aldh1a2* expression and sex steroid signaling is relevant for the regulation of gonopodium formation or growth, we over-activated the RA signaling pathway. In gonopodial rays (3–4–5 complex), intraperitoneal (IP) injection of RA resulted in a significant increase in the gonopodial growth rate (Fig. 8B) with a strong effect on the segmented rays that are laid down proximally (Fig. 8C) and milder effects on the size of the proliferating region in the distal tips of fin rays (Fig. 8D).

The effects of RA on the increase of the growth rate (Fig. 8B) and on the size of the proliferating distal tip (Fig. 8D) are specific to gonopodial rays since no significant changes were found in non-gonopodial rays (ray 6, 7 and 8) of RA-injected fish compared to control fish (Fig. 8). Although significant effects on segment length (Fig. 8C) were found in both gonopodial and non-gonopodial rays, the amount of increase in gonopodial rays (rays 3 and 4) was much greater than in non-gonopodial rays (rays 7 and 8). Ray elongation in RA-injected fish compared to control fish was 239 µm (ray 3), 147 µm (ray 4), 33 µm (ray 7) and 23 µm (ray 8), respectively (Fig 8C). It is interesting to note that the non-gonopodial ray 6 retains high expression of *rar-ga* that is comparable to r3–5 (Fig. 4E) and can be “forced” to at least form a few additional segments in a situation of artificial episodic testosterone exposure (Fig. 7). It thus appears as if r6 lacks so far unknown signals that would be required for sustained growth and inclusion in the gonopodium. In summary, RA treatment significantly enhances growth of the 3–4–5 complex, whereas growth in non-gonopodial rays is comparatively moderate. However, we could not completely rule out the possibility that RA might have effects on outgrowth of non-gonopodial rays that we did not detect during the experiment.

It has recently been shown that enhanced RA signaling promotes mesenchymal proliferation during regenerative growth of the zebrafish caudal fin [41]. Specifically, RA signaling is essential for the proliferation of cells in the blastema, which is located in the distal tips of regenerating rays, by cooperating with Fgf, Wnt/β-catenin and non-canonical Wnt signaling pathways. Our findings suggest that RA signaling fulfils a role in promoting the proliferation of cells in the distal fin ray mesenchyme both in development and regeneration.

The increase in the number of new segments upon RA injection was strong and significant only in the gonopodial rays (Fig. 8A). This might suggest that RA plays a role in fin segmentation and acts more specifically in gonopodial rays (Fig. 8A). Together with the increase in segment numbers by increased RA signaling, this treatment also produces slightly smaller segments (reduced by 18% in RA-injected fish compared to controls; data not shown). Although a direct comparison of average segment sizes requires caution, because control fish produced fewer segments than RA-injected fish, these results imply a potential role for RA signaling in the segmentation process. This could be important for the formation of the small segments that characterize the distal region of naturally developing gonopodia (Fig. 1B). Interestingly, the mechanism by which RA determines the proximo-distal axis of the developing limb is reactivated during limb regeneration [60]. Altogether, RA signaling influences the proximodistal patterning not only in embryonic development but also in post-embryonic metamorphosis like developing gonopodia or fin regeneration.

### Interaction between androgen and RA signaling during gonopodia development

Our study shows a link between androgen and RA signaling during gonopodium development and suggests that some of the effects of testosterone are mediated by an increase in *aldh1a2* expression and concomitant RA signaling. Gonopodium development is activated by elevated levels of testosterone in poeciliid fish [8], [12], [13], [61] and we show that elevated levels of testosterone induce further developed gonopodia. Several molecular studies support this view: (1) expression of genes involved in fin ray growth, such as *msxC*, *fgfr1* and *shh*, is induced by testosterone [9], [19], [20]. It has been suggested that androgen signaling acts upstream of a signaling cascade that results in the activation of downstream effectors, such as Fgf-signaling and *msxC*, to shape the gonopodium [9], [20], (2) *androgen receptors* (*ars*) are expressed in the developing gonopodium (this study and [19]) and (3) inhibition of androgen signaling down-regulates gene expression and perturbs gonopodium growth [19]. We show that *aldh1a2* expression is induced by testosterone in the anal fin (Fig. 4) and its levels are correlated with the concentration of testosterone (Fig. 6A), suggesting that androgen signaling regulates *aldh1a2* expression. We show that expression of *arb* overlaps with that of *aldh1a2* in the distal-most mesenchyme of the 3–4–5 complex and also in the anal fin rays 6 and 7. The *arb* gene is expressed in the segment boundaries of two adjoining segments in the gonopodium, which only becomes visible after prolonged staining (data not shown). The function of *arb* in the segment boundaries is unknown, but it is possible that *arb* is directly involved in the establishment or maintenance of segment boundaries. Under this scenario increased levels of testosterone and RA would promote fin ray segmentation, which is supported by the outcomes of RA injection experiments. In addition, a role of *ara* in *aldh1a2* regulation cannot be ruled out, since *ara* is also expressed in the developing gonopodium, though in a diffuse pattern. *arb* expression also overlaps with that of the two *rar-g* paralogs. Experiments in rats confirmed a regulation of *rar-g* by androgen signaling in several tissues [62]. Several studies suggest that there may be crosstalk between RA- and sex steroid signaling pathways during embryonic development of sexual organs or reproductive systems, e.g., in the development of imposex in gastropod females [63], the development of mammalian external genitalia [64] and sexually dimorphic anal fin, which is an electric discharge organ in electric fish [65].

We propose that the sex steroid-controlled metamorphosis of the anal fin towards a gonopodium is mediated by an increase in RA signaling. In support of this idea, we show that *arb* (and *ara*) expression overlaps with *aldh1a2* and *rarg* expression in the growing tips of anal rays that are eventually incorporated into the mature gonopodium. Importantly, increased androgen signaling in artificially and naturally induced gonopodia significantly up-regulates *aldh1a2* expression (RA synthesis) and increased RA signaling in artificially induced gonopodia dramatically improves proliferation and fin ray segmentation in the gonopodium. The molecular mechanisms of gonopodium development are thought to be evolutionarily conserved, despite some variation in its morphology (e.g. terminal structures) between species [6], [13]. RA is a well-known key morphogen during embryonic development and our work highlights a role for RA signaling in post-embryonic metamorphosis.
